# CORHOH: Text corpus of holocaust oral histories

**DOI:** 10.1016/j.dib.2025.111426

**Published:** 2025-02-24

**Authors:** Daban Q. Jaff

**Affiliations:** Faculty of Philosophy, University of Erfurt, Germany

**Keywords:** Corpus, Linguistics data, Oral histories, Holocaust Survivors, Text of Oral Histories, Digital humanities, Genocide narratives

## Abstract

This paper outlines the compilation and annotation process of CORHOH: Text CORpus of **H**olocaust **O**ral **H**istories. The corpus consists of 500 oral histories, each narrative form one survivor. The transcripts of the oral histories are retrieved from the *Let Them Speak Project* [1]. The transcripts are normalized and further annotated. The corpus offers rich metadata about both the testimony givers and the interviews. All technical content is removed, and a unique identifier is assigned to each question (posed by the interviewer) and answer (provided by the survivor). The corpus complies with the TEI guidelines [2]. The corpus includes 106,519 questions and 107,125 answers, making it easy to distinguish between the utterances that belong to the holocaust survivor or anyone else who is involved in the interview, primarily the interviewer. CORHOH is particularly suited for studies on trauma expression and psychological concepts embedded in survivors' narratives. Additionally, it offers potential for data mining to uncover patterns (e.g., migration trends) and supports natural language processing techniques, such as topic modelling, sentiment analysis, and named entity recognition. The CORHOH data is courtesy of the United States Holocaust Memorial Museum (USHMM) and is publicly available under the CC BY-NC-SA 4.0 license.

Specifications Table

**Table:** 

Subject	Social Sciences
Specific subject area	*Corpus. Holocaust oral histories. Linguistics data.*
Type of data	*xml, xsd*
Data collection	The following steps are involved in data collection and processing: 1. Metadata Creation: Information is gathered on each testimony giver, including their name, date of birth, gender, birthplace, whether they experienced a ghetto or camp, immigration details, and the interview files, including date of recording, the USHMM unique code, type of permission. 2. Data Retrieval: The raw text of each oral history transcripts is retrieved from the Let Them Speak Project [1]. 3. Data Processing: Texts are normalised and annotated, ensuring all metadata is accurately linked to the corresponding oral histories.
Data source location	The data originates from the Let Them Speak [1], which integrates contributions from: 1. The Fortunoff Video Archive for Holocaust Testimonies (FVAHT): https://fortunoff.library.yale.edu 2. The USC Shoah Foundation: https://sfi.usc.edu/ 3. The The USHMM: https://www.ushmm.org The CORHOH corpus exclusively includes oral histories from the USHMM.
Data accessibility	Repository name: Mendeley Data identification number: DOI:10.17632/gz7v268252.2 Direct URL to data: https://data.mendeley.com/datasets/gz7v268252/2
Related research article	Not applicable

## 1 Value of the Data

• **Preservation of Survivor Oral Histories**: The corpus offers a comprehensive collection of first-hand Holocaust oral histories. Also, it offers an efficient way of preserving the text of oral histories for the next generations.
• **Rich Metadata**: The corpus offers rich and detailed metadata on each testimony giver and oral history which could enable interdisciplinary research, including studies on history, sociology, and cultural anthropology.
• **Linguistic and NLP Applications**: The corpus is a resource for examining how survivors, employing language, including linguistic devices like pauses, hedging, figurative devices, and repetition to express their emotions. It is also well-suited for advanced natural language processing tasks, including sentiment analysis, topic modeling, and keyword frequency analysis.
• **Psychological Insights**: By capturing narratives imbued with memories of war, persecution, and resilience, the corpus supports studies exploring the long-term psychological effects of trauma and the coping mechanisms employed by survivors.

## 2 Background

CORHOH is compiled as part of a PhD project focusing on Holocaust oral histories, particularly the figurative language adopted by survivors to express their experiences. The project examines whether survivors employ different linguistic devices to convey traumatic emotions versus mundane memories and seeks to understand the emotional depth of these narratives. On the other hand, despite global efforts to remember the Holocaust atrocities—especially as the 80th Holocaust Remembrance Day is commemorated in 2025, there remains a significant gap in comprehensive, centralized repositories of raw Holocaust oral history texts. Existing initiatives [[1], [2],^3^] are pioneering works representing significant efforts in analyzing Holocaust oral history texts, providing valuable insights into survivor narratives. However, they often do not offer access to the complete raw text, detailed metadata, or unified annotation to distinguish between the parts of the text that belong to the testimony giver and those that do not. To address this, CORHOH consolidates a large number of oral histories in an accessible, well-structured format. By making this resource available, it aims to support researchers, and the public in preserving and analyzing Holocaust oral histories, thus contributing to a deeper understanding of this history.

## 3 Data Description

**Fig. 1:**
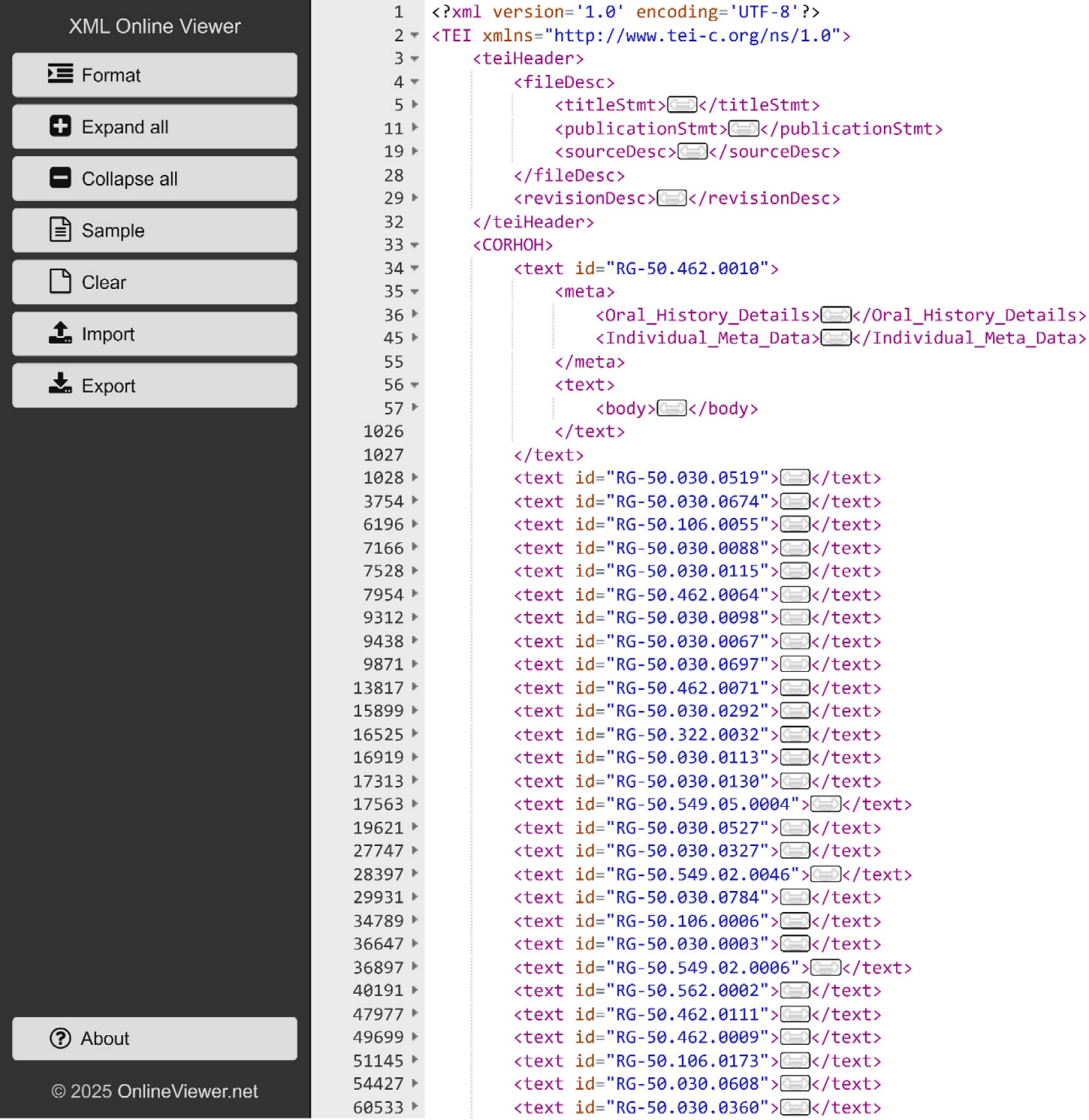
The corpus structure, as depicted in the screenshot. https://xml.onlineviewer.net/.

The corpus is in XML format and follows a hierarchical structure; the structure is fully described in complementary xsd file. This structure is consistent across all records in the corpus. The xml file is hosted under the main root < **TEI xmlns**>, Fig. 1:

**1**. **<teiHeader>** is the element that contains metadata about the corpus.
**2**. **<CORHOH>** is the second element; it nest 500 elements (each for an oral history record, (e.g., < **text id="RG-XX.XXX.XX.XXXX>** or **< text id="RG-XX.XXX.XXXX>)**:

**2.1 <text id="RG-XX.XXX.XXXX"> (per Record)**: Each oral history record is typically identified by a unique identifier (e.g., <RG-XX.XXX.XX.XXXX> or <RG-XX.XXX.XXXX>) which corresponds to the USHMM's cataloging system as follows:

**2.1.1 <meta>**: it hosts two sections.

I. **<Oral_History_Details>**: Includes details about the oral history:
  ■ Documents_ID: The unique identifier for the record.
  ▪ Rec_Date: The recording date.
  ▪ Rec_Length: The duration of the recording.
  ▪ A_Number: Sequence of answers.
  ▪ Q_Number: Sequence of questions.
  ▪ permission_type: Indicates the access and usage permission (e.g., "No restrictions").
  ▪ Link: URL to the record in the archive.
II. **<Individual_Meta_Data>**: Contains personal metadata about the interviewee:
  ▪ Name: Full name of the testimony giver.
  ▪ DOB: Date of birth.
  ▪ Gender: Gender of the testimony giver.
  ▪ Born: Place of birth.
  ▪ Ghetto: Ghetto (if applicable).
  ▪ Camp: Camp (if applicable).
  ▪ Imm_Date: Immigration date.
  ▪ Imm_Destination: Immigration destination.

**2.1.2 <text>**: This section contains the verbatim transcript of the oral history interview that corresponds to the metadata preceded. The structure is as follows:

i. **<text>**: Contains the entire body of the interview.
ii. **<body>**: The core content of the interview.
iii. **<div>**: representing the type of text (“interview”).
iv. **<head>**: Headings or titles for sections ("Interview Transcript").
v. **<div>**: Nested sections within the body containing individual dialogue exchanges. This occurs for each utterance as follows:
  A. **<speaker>**: Labels the speaker ("Interviewer" or "Interviewee"), in addition to type of the utterances and it is unique annotation in reference to the corpus (“Axxx” or “Qxxx”).
  B. **<u>**: Represents the utterances or dialogue spoken by the speaker, in question-answer format.

## 4 Experimental Design, Materials and Methods

### 4.1 Metadata creation

The process begins with gathering detailed information about testimony givers to form the metadata. The oral histories in this corpus are selected based on the following inclusion criteria:

**A**. Age Requirement: only oral histories of testimony givers who were at least 4 years old before the outbreak of World War II are included in the corpus. This criterion ensures that all testimony givers had direct memories or experiences of that period [4].
**B**. Immigration Context: the corpus only includes oral histories of the testimony givers who immigrated to English-speaking countries (e.g., the United States, Canada, Australia, or the United Kingdom) to focus on oral histories available in English.

#### 4.1.1 Primary sources of metadata

Metadata about oral histories is primarily retrieved from the USHMM website. As for the testimony giver, the following sources are consulted:

**A**. USHMM
**B**. Let Them Speak Project Website [1]
**C**. Oral histories
**D**. When a piece of metadata could not be found from the primary sources above, additional archives and resources are consulted to ensure completeness and reliability.

### 4.2 Data retrieval

After finalizing the metadata, the raw text of oral histories is retrieved from the Let Them Speak Project website [1] using unique identifiers. These identifiers ensured the accurate matching and retrieval of relevant files.

### 4.3 Data processing

The major task in this step is to identify what belongs to testimony giver and what belongs to others. Then, the second objective, unifying the paragraph tags across the corpus as follows:

• Q: Represents questions asked during the interviews.
• A: Represents answers provided by the testimony givers.

To achieve these objectives, the retrieved data undergo several steps:

**A**. **Paragraph tagging analysis**

In this step, a python script returns the tag of the paragraphs in the corpus, Table 1. Before normalization, there are 221,893 instances, with 165,894 correctly annotated, accounting for 74.8% accurate annotations. The noise level is 8,249. After normalization, the total count is adjusted to 213,644.

**B**. **Unifying annotations across the dataset**

i. Normalization, step one: after removing timestamp, noises, footnotes, technical conversation (such as tape #). “A., A” and “Q., Q” are replaced with “A: ” and “Q: “ with RegEx function in Sublime Text across the dataset. At the end of this step, 167,304 paragraphs, accounting for 78.31 %, are correctly tagged.
ii. Normalization, step two: A script returns paragraph tags and their ending as follows:
  For example, ‘FW’ (paragraph annotation): Total: 183(number of the annotation in the corpus)

Percentage of paragraphs that annotated with ‘FW’ ending with question mark: 83.61 %
Percentage of paragraphs that annotated with ‘FW’ ending with period: 15.30 %
Or
IH: Total: 150
Percentage Ending with Question Mark: 0.67 %
Percentage Ending with Period: 96.67 %
When the percentage of question mark ending is higher, the annotation is deemed to be interviewer and the annotation are replaced with “Q: ”, such as “FW”. When the percentage of ending with period is higher, the annotation deemed interviewee and the annotation is replaced with “A: ”, such as “IH”.

iii. When the percentage is close or not indicative, the document number is retrieved and checked manually to annotate the paragraphs properly. Also, some of the returned tags are full wors which is indication that the paragraphs in some of documents are not annotated. In such cases, the document is examined manually for proper annotation.

**Table 1:** Top 10 most frequent tags used in the beginning of paragraph in the dataset.

Rank	Word	Frequency
1	Q:	83,927
2	A:	81,967
3	JF:	3835
4	NL:	2429
5	EM:	2279
6	SS”	2188
7	A.	1708
8	Q.	1702
9	HF:	1176
10	MF:	1117

## Limitations

CORHOH's primary limitation is its restriction to English-language oral histories. Future work can focus on compiling a multi-language oral history corpus to broaden its scope and accessibility. Additionally, this corpus includes an oral history per firsthand holocaust survivor. Many Holocaust survivors have provided multiple oral histories, and future efforts could address this by hosting more than one oral history from the same individual in a unified corpus. As we commemorate the 80th Holocaust Remembrance Day, it is important to recognize that at least two subsequent generations—the second and third—have also been profoundly impacted by the Holocaust in various ways. Future work can focus on collecting, categorizing, and digitizing oral histories from these generations to provide a more comprehensive understanding of the Holocaust's long-term effects. Lastly, while the metadata in this corpus is extensive, there are limitations in obtaining certain details, such as the immigration dates, for example, for some testimony givers. Despite best efforts, some of these entries remain incomplete and their value return “nan” in CORHOH.

## Ethics Statement

All data used in this study comply with ethical guidelines of the United States Holocaust Memorial Museum (https://www.ushmm.org/copyright-and-legal-information/terms-of-use), and the oral histories included in the CORHOH corpus are publicly available under the CC BY-NC-SA 4.0 license.

## Data Availability

Mendeley DataText Corpus Of Holocaust Oral Histories (Reference data).
